# Charlie Gard and the weight of parental rights to seek experimental treatment

**DOI:** 10.1136/medethics-2017-104718

**Published:** 2018-05-17

**Authors:** Giles Birchley

**Keywords:** quality/value of life/personhood, minors/parental consent, rights, children

## Abstract

The case of Charlie Gard, an infant with a genetic illness whose parents sought experimental treatment in the USA, brought important debates about the moral status of parents and children to the public eye. After setting out the facts of the case, this article considers some of these debates through the lens of parental rights. Parental rights are most commonly based on the promotion of a child’s welfare; however, in Charlie’s case, promotion of Charlie’s welfare cannot explain every fact of the case. Indeed, some seem most logically to extend from intrinsic parental rights, that is, parental rights that exist independent of welfare promotion. I observe that a strong claim for intrinsic parental rights can be built on arguments for genetic propriety and children’s limited personhood. Critique of these arguments suggests the scope of parental rights remains limited: property rights entail proper use; non-personhood includes only a small cohort of very young or seriously intellectually disabled children and the uniqueness of parental genetic connection is limited. Moreover, there are cogent arguments about parents’ competence to make judgements, and public interest arguments against allowing access to experimental treatment. Nevertheless, while arguments based on propriety may raise concerns about the attitude involved in envisioning children as property, I conclude that these arguments do appear to offer a prima facie case for a parental right to seek experimental treatment in certain limited circumstances.

## Introduction

The case of Charlie Gard concerned the right of parents of an infant with a genetic illness to take him to the USA to receive experimental treatment against medical advice. In July 2017, a spokesperson for the family announced that frustrating this right amounted to Charlie being ‘taken prisoner by the NHS’.^1^ In this article, I consider the basis and scope of parental rights (PR) in relation to the details of Charlie’s case, primarily drawn from the legal records.^2–6^ Discussion of moral rights is justified here because these should be accounted for in future cases. Further, moral rights can translate to legal rights, and thus may ultimately compel particular decisions. I argue that, although the case was decided on welfare grounds, there is evidence of attention to rights of Charlie’s parents that are independent of a welfare basis—what I shall term ‘intrinsic PR’. Exploring the basis of such rights, I suggest that the strongest philosophical argument for PR is that Charlie was a non-person and genetically his parents’ property. These rights are strictly limited, and in Charlie’s case PR were outweighed by a precautionary approach to the uncertain harms accruing to Charlie. However, in future similar cases, PR may prove determinative where harms are excluded and potential benefits are great. I argue that parents are competent to exercise these rights, and that public interest concerns over access to experimental treatment can be allayed.

## Charlie’s case

Charlie was born with infantile-onset mitochondrial DNA depletion syndrome (MDDS). MDDS is a genetic disease where abnormal mitochondrial DNA causes cells to malfunction. Different strains of MDDS cause variable levels of disability. Charlie’s rare ‘RRM2B’ mutation of MDDS caused progressive brain and muscle damage.

Charlie was admitted to hospital at 2 months. A ventilator helped him to breath and he had recurrent seizures. He could not move his limbs and opened his eyes only intermittently, making it difficult to tell when he was in pain. Charlie’s parents became aware of experimental treatment (nucleoside therapy), where biochemical food supplements stimulate the repair of mitochondrial DNA. Results of nucleoside therapy on mice with the TK2 variant of MDDS (which usually leaves the brain unaffected) increased lifespan slightly. Human trials saw 13 (of 18) children with TK2 MDDS grow, and one improve their walking ability over 4 years. There was no evidence the therapy repaired the brain, nor was it tried on RRM2B MDDS. Nevertheless, a US expert claimed it might help Charlie.

Charlie’s doctors agreed to try the therapy. However, before a trial commenced, Charlie suffered several weeks of refractory epileptic fits. This caused severe brain damage and Charlie’s doctors believed he had no hope of improvement, counselling fatal withdrawal of ventilation. Charlie’s parents remain steadfast in their wish for further treatment, including nucleoside therapy. The hospital sought a court order that it would be lawful to withdraw treatment because treatment was not in Charlie’s best interests. Charlie’s parents argued that they knew Charlie best, and cared for him more deeply than anyone else. Yet, apart from Charlie’s parents and the clinician offering treatment, all witnesses agreed with Charlie’s doctors. The order the hospital sought was eventually granted, and affirmed in three subsequent appeals.^2–5^ A final spate of litigation settled when and where withdrawal would take place. Charlie’s parents bitterly complained that the outcome left them ‘very little time’ before Charlie died.^7^ Withdrawal did not take place immediately, but after an unspecified period of time set out in a confidential annexe to the court order.^6^ Evidently, this was brief as reports of Charlie’s death were published after 18:00 the following day.^8–10^

## PR and the limitations of welfare

Broadly speaking, a right may be seen as a way of empowering an individual against collective goals that disregard that individual’s legitimate interests.^11^ Moral rights are normative claims supportable by reasoned arguments. Legal rights can be (and often have been) argued for on the basis of a putative moral right.^12^ Legal rights may impose binding duties on others to either enable or, more commonly, not to impede the exercise of that right. The potential to influence the actions of decision-makers makes it pertinent to consider moral rights when assessing whether Charlie’s case should alter our attitudes to the parental role in decision-making in similar cases.

In disputes about children’s care ‘[m]any of today’s critics of children’s rights are passionate defenders of … the rights of parents’^13^: different ethical approaches to children and parents make asymmetric claims about the putative moral rights of both parties. Arguments about rights may suggest that children: have rights; have rights that others (especially parents) should articulate; do not have rights. That parents: have rights based on their ability to advance the welfare of their child(ren); have rights over their children that are intrinsic to their status as parents. Broadly speaking (and leaving aside thornier issues of competence), my own considered moral judgement is that decisions concerning children who are unable to articulate their own wishes should be attentive to the child’s welfare. In other words, PR are derivative of their ability to advance the welfare of their child. This view is shared by many^14–16^ even if they disagree about the scope of such rights or how welfare should be measured. In Charlie’s case, welfare was a key factor for the courts. All sides (including the clinician proposing to deliver the innovative therapy) agreed that the chances of Charlie benefiting from treatment were ‘vanishingly small’.^7^ Yet, while a focus on welfare suggests that the only basis of PR in this case was Charlie’s welfare, some unremarkable aspects of the case do not comfortably fit this welfare thesis. In particular, the decision to delay withdrawal of treatment for a brief period after the courts had reached their decision, however appropriate we might feel such behaviour is, clearly continued whatever harms Charlie was experiencing. Delaying withdrawal is not unusual in similar cases.^17^ Delays are likely to be motivated, at least in part, by consideration of the interests of Charlie’s parents rather than resting exclusively on Charlie’s parents’ ability to advance Charlie’s welfare. To coherently explain why parents should be owed such respect, and to explore whether a similar basis suggests PR to have demands for experimental treatment met, it is important to consider the philosophical basis of intrinsic PR. The next section explores some of these arguments.

## Intrinsic PR

Arguments suggesting parents have an intrinsic right to be decision-makers for their children include the argument that parenting has a teleological basis and is a good in itself.^18 19^ Other arguments are based on the child being parental property, termed ‘proprietarian’ accounts.^12^ I consider the latter to contain the strongest arguments for PR. Therefore, after briefly dismissing a teleological account, my discussion concentrates on these.

While teleological accounts vary, Page^18^ offers a particularly rich account. It asserts that both procreation and rearing children are essential parts of being human, and thus a good in themselves. PR, Page argues, are like sexual rights; restriction of either offends human nature. Page’s arguments more strongly assert a right to procreate (eg, against parental licensing)^20^ than a right to rear. While a teleological account depends on the optimal fulfilment of human nature, known as ‘flourishing’, it is by no means clear that having children is essential to flourishing. Indeed, depending on how flourishing is construed, it may be salient to consider that having children may not be fulfilling for parents,^21^ nor that adults without children lack fulfilment. The status of parenting as a good in itself is open to question. More promising than teleological accounts are proprietarian accounts. Although thorough-going proprietarian arguments are rare, inclinations towards proprietarianism may be more widespread. For example, some detect proprietarianism in Nozick’s influential libertarian philosophy.^12 22^ Indeed, English common law has been argued to implicitly recognise a property right in the child.^23^

Proprietarian accounts^24 25^—including that of the bioethicist H Tristram Engelhardt^26^—have a common root. They use claims made by John Locke^27^ about the nature of property and self-ownership as a basis for proprietarianism.^26^ Locke’s first claim is that producers have rights of ownership over the products their labours create. According to Locke, these ownership rights are gained by ‘mixing’ the producer’s labour with the product. Locke himself argued that children were not parental property. Some find Locke’s reasons for excluding children—that they are owned by God, not parents—unconvincing,^28^ and apply his arguments to children nevertheless.^25 26^ Yet the ‘mixed labour’ argument is a problematic source of PR, implying that anyone who makes efforts affecting a child has rights over that child,^12 29^ and struggling with parental equality.^12 30^ Locke’s second argument is that people own themselves. This is commonly^29 31^ tied to claims that PR stem from their children being (in some sense) part of the parent’s bodies. Some think this could only realistically apply to pregnant women.^12 32^ Others argue that ownership follows parental provision of the child’s genetic material^25^ or genetic information.^33^ These arguments suffer a regress problem. Parents were children once and logically, parents must still be owned, in turn, by their own parents. To overcome the regress, children must sometime stop being parental property. However, genetic *material* is rapidly subsumed in a sea of new cells, meaning genetic propriety is lost early in gestation, while genetic *information* lasts a lifetime and so remains susceptible to the regress.^33^ However, the regress may be overcome if dwindling ownership is based on emerging personhood.^26^

Personhood is a way of distinguishing morally important, and morally less important, beings. One account of personhood, from Singer,^34^ argues that being human is not a morally relevant property. Instead he argues that moral status is conferred by being a *person*. A person in Singerian terms must be capable of conscious introspection and a sense of futurity. Thus, some animals will be persons (eg, chimpanzees) while some genetic humans are non-persons (eg, anencephalic infants). Although suffering to non-persons is morally undesirable, non-persons are less morally important than persons.^35^ Engelhardt links children’s putative non-personhood to proprietarianism, asserting children lack the faculties necessary to be persons, and thus start as the moral inferiors of their parents.^26^ As children mature, they attain personhood and become morally equivalent to their parents. Such an account overcomes the regress problem. I suggest an account on PR based on this reasoning, prima facie, fairly strong. In the next section of this article, I critique this account to determine some limits of intrinsic PR.

## The limits of intrinsic PR

I have postulated a basis for intrinsic PR relies on two key elements: genetic propriety and children’s non-personhood. The scope of PR depends on the strength of these elements.

An obvious objection to this account is that children ought not to be described as property. An extraordinary range of commentators take pains to emphasise this position.^15 36 37^ One basis given for this claim is that it harms children to be treated as property.^29^ Yet proprietarians argue that parents are still be bound to treat their children non-malevolently.^26^ Restrictions against misuse are a well-argued feature of legitimate property rights, including an obligation not to use property to harm others.^38^ Lyons^23^ argues that misuse of children could be prohibited by classing them as inalienable property, and this suggests the obligation against harm could extend to the child themselves. Argued this way, some consider the consequences of proprietarianism are proximate to the welfare view of PR.^30^ While describing children as property may evoke concern, it is difficult to devise a convincing argument against PR founded solely on this basis. Nevertheless, we should note that the scope of proprietarian PR is restricted, although based on avoiding harm and misuse, rather than promoting welfare.

The proprietarian account appears more vulnerable in claiming that children are non-persons. The non-personhood claim coheres with widespread public intuitions that the life of a fetus is less morally important than the woman who carries it.^39^ Without consideration of the moral status of fetuses here, these intuitions at least suggest arguments using personhood should be seriously considered when asserted in relation to older children. However, even if^40 41^ we accept that personhood is morally legitimate, it is arguable that under common personhood criteria, most children are persons, suggesting further limits to the scope of PR. Based on criteria of futurity and self-awareness, Singer^34^ argues children in the neonatal period (<1 month) are non-persons. Ross^42 43^ suggests that children up to late adolescence are not capable of rational decisions and thus ‘not full Kantian persons’. There is evidence that disputes these assumptions. Neonates appear to anticipate familiar events^40^ and distinguish their own touch^44^ and cry.^45^ This suggests neonates may possess a sense of futurity and self-consciousness—meeting Singerian criteria for personhood. Further, interviews with chronically ill children and their clinicians suggest that children as young as 3 years old may be capable of making rational decisions,^16^ challenging Ross’ claims. On this basis, parents’ rights over their children arguably begin waning between a few days and a few years after birth. However, PR may be much more persistent in children who are severely intellectually disabled, like Charlie.

A final criticism of proprietarianism, which may further reduce the scope of PR, is based on criticism of genetic propriety. If parental property rights stem from parental ownership of the child’s genetic information, it appears to support the claim that genetically related parents should have a greater say in decisions about their child than non-genetically related parents, such as adoptive parents. Yet it seems unconvincing to suggest this when the genetic parents have no interest in, care for, or knowledge of, their child. Even if we disregard these objections, genetic similarity seems a fragile basis on which to base *exclusively parental* rights. Although familial resemblances are commonly cited as important flags of identity,^46^ it is by no means clear that these socially constructed meanings rest on genetic resemblances rather than being proxies of social connection.^47^ The global population has extensive genetic similarity^48^: otherwise unconnected individuals have vastly more genetic information in common than they do not.^49^ Genetic propriety accounts rest on a very slender material basis for such a connection between the child and the parent. Indeed, taken proportionately, it may also imply a shared genetic propriety of children. If the proportion of unique parental property in children is discernible, but very small, perhaps the rights of parents that can derive from this property are proportionately small.

These arguments do not suggest that intrinsic PR cannot be made out. However, the scope of these rights is restricted. By being based on a property right they limit the PR to non-harmful acts. On the most generous account the emergent personhood of the child causes intrinsic PR to expire after a few years in most cases. Finally, intrinsic PR based on genetic propriety are little greater than the rights of unrelated humans. These tangible, but weak, reasons for intrinsic PR may support transient delays in securing their child’s welfare. However, they are unlikely to be strong enough to allow parents to undertake prolonged acts against their child’s well-being.

## Looking to the future: PR to access to experimental treatment

Given the justifiable, but slim, bases for PR, one of the considerations to arise from Charlie’s case is to ask under what circumstances PR could influence future cases about accessing experimental treatment. Having established that PR beyond those based on enhancing the welfare of the child are both weak and limited, crucial to such questions will be the harms and benefits likely to arise from experimental treatment. The experimental basis means benefits will be uncertain. Indeed, a defining feature of potentially fatal cases involving critically ill children is the disagreement about whether the same outcome is a harm or a benefit. On one side, living with intensive care may be understood to amount to harm, and death a benefit^50^; on the other side, loss of life is the greatest harm, and continued intensive care is a benefit. This disagreement is additionally complicated by the uncertain outcomes of experimental treatment. In Charlie’s case, the risks of experimental treatment alone (a food supplement) were inconsiderable. Intrinsic PR combined with uncertain and minimal potential benefit (because of Charlie’s brain injuries) were too weak to overcome the presence of uncertainty about harms, and a precautionary approach was taken. By precautionary approach, I mean broadly, the exercise of the principle of precaution, where uncertainty about the harms and risks that arise from a technology new to science means we should err on the side of caution. While I lack space to offer detailed argument, such an approach has been cogently argued to be pertinent to the regulation of innovation in healthcare^51^ and neonatal non-treatment.^52^ Yet the ability to overcome reservations about uncertainty in pursuit of potentially great benefits may be a feature of intrinsic PR. In future cases where harm is minimal and benefits are uncertain, but potentially great, intrinsic PR may be determinative. However, there are other objections to accessing experimental treatment. First, are parents competent to make these judgements? Second, are there public interests grounds on which to deny competent demands?

If parents have rights to demand experimental treatment, an additional concern is whether they can rationally exercise these rights. Questioning the ability of terminally ill persons to make rational judgements about experimental treatment, Caplan argues that terminally ill people are likely to view any experimental treatment with undue optimism.^53^ The moral weight of these elements changes where the patient is a child. The question of *parental* competence to decide is a thorny one. The challenges to parental competence are clear if we consider Charlie’s case, where Charlie’s parents were vulnerable to manipulation by third parties.^54^ These emotional challenges lead some to question the ability of parents to offer meaningful consent in life and death situations.^55^ Yet the enormity of parental bereavement suggests that the degree of parental incompetence should be extreme indeed, if it is to over-rule a parental right of say (in practice, this circle is of course squared by the current legal process, which allows the courts to make a decision if parents and clinicians disagree).^56^

Bender *et al*^57^ offer public interest arguments for restricting access to experimental treatments. They argue that premature access to experimental treatments may raise false expectations among those with similar illnesses, igniting public demand. If this demand is not resisted, drugs may be made widely available before their effectiveness is proven, leading to potentially ineffective treatments becoming the standard of care. The authors also argue that early access to unproven drugs may both hamper recruitment to, and reduce industry incentives to run, clinical trials. First, recruitment is hampered because early access physically reduces the number of potential drug trial participants. Second, if drug companies are paid for access unproven drugs, it provides a commercial disincentive for clinical trial sponsorship. Not only are trials costly and time consuming, but the fact they risk jeopardising a proven source of profit if the experimental treatment proves ineffective provides a clear conflict of interest. These arguments suggest that, whatever the benefit to the patient, early access has opportunity costs, because inadequate knowledge of effectiveness denies future patients access to treatments with a proven basis. Ultimately there is a price to pay beyond the risks and benefits to an individual patient. However, preventing the exercise of individual choice to protect public interests may appear to be a paradigm case of group interests impinging on individual interests. This is what rights are designed to prevent. Certainly, it has been argued that where patients seek innovative lifesaving treatments a rights approach requires that any prohibition on treatment must be undergirded by extremely weighty risks if they are to defensibly outweigh the patient’s right to life.^58^

The choice of patients who are terminally ill to access treatment may carry enough moral weight to overcome objections in the public interest. The weakness of PR alters the dynamics of such choices when the choice is a parental one. My analysis suggests that in a future case intrinsic PR could overcome uncertainty in the face of large potential benefits. Certainly, the English courts have in the past allowed access innovative treatment in such circumstances without an obvious prohibition in the public interest.^59^ Indeed, with the right approach these factors could be mitigated further in a future similar case to Charlie’s. There is little prospect of clinical trials on a rare mutation like RRM2B. Early access linked to a robust, if novel, trial design may accelerate understanding of the experimental drug, and thus allay public interest concerns. It is notable in Charlie’s case that no evidence was advanced that access was to be part of such a trial. Public interest concerns may therefore have placed additional obstacles in the way of PR in Charlie’s case.

## Conclusion

Charlie’s case brought questions about the moral status of parents and children into the public eye. The case apparently fell on welfare grounds; however, not every fact of the case is consistent with the promotion of Charlie’s welfare. Reviewing the grounds for intrinsic PR that are independent of the welfare of their child, I suggest the strongest of these lie in arguments for genetic propriety and children’s non-personhood. Critiquing these claims suggests limits to intrinsic PR. Nevertheless, I argue that these claims provide grounds to accept PR for experimental treatment in future cases involving children who cannot contribute an opinion. These cases must involve no harm to the child and benefits, despite being uncertain, must be potentially great. Further, I argue parents are competent to exercise these rights, and that objections in the public interest may be overcome by evidence of robust trial design in the experimental treatment.
